# Microstructure and Mechanical Properties of Laser-MIG Hybrid Welding of Invar36 Alloy with Different Grooves

**DOI:** 10.3390/ma18225066

**Published:** 2025-11-07

**Authors:** Dehao Kong, Shiwei Zhang, Hong Bian, Jun Tao, Yang Dong, Xiaoguo Song, Caiwang Tan

**Affiliations:** 1 Aeronautical Key Laboratory for Welding and Joining Technologies, AVIC Manufacturing Technology Institute, Beijing 100024, China; 15249881983@163.com (D.K.);; 2 State Key Laboratory of Precision Welding & Joining of Materials and Structures, Harbin Institute of Technology, Harbin 150001, China; bianhong@hit.edu.cn (H.B.); xgsong@hitwh.edu.cn (X.S.);; 3 Shandong Provincial Key Lab of Special Welding Technology, Harbin Institute of Technology at Weihai, Weihai 264209, China

**Keywords:** Invar36 alloy, laser-MIG hybrid welding, welding grooves, microstructure, mechanical properties, finite element method

## Abstract

Laser-MIG hybrid welding experiments were performed on 10 mm thick Invar36 alloy plates. The influence of three different types of welding grooves (V-shape, rectangle, and X-shape) on the microstructure and mechanical properties of the welded joints were analyzed. The results indicated that the grain growth morphologies and grain sizes varied among the grooves. The average grain size at the center of the weld seam was 177.97 μm, which was smaller than the top grain size of 317.29 μm and the bottom grain size of 233.59 μm. In V-shape and rectangle grooves, the dimensions of grain the first weld pass was obviously smaller than the top region of the second pass. Microstructural characterization and tensile test showed no vertically columnar grains along the weld centerline in rectangle grooves which significantly affected the mechanical properties of welded joints. As a result of this phenomenon, V-shape groove joints demonstrated better mechanical properties than rectangle groove joints. The highest average tensile strength for V-shape groove, X-shape groove, and rectangle groove joints were 429.0 MPa, 419.3 MPa, and 395.4 MPa, respectively. Based on the Abaqus software, three-dimensional finite element analyses of three groove types were performed to investigate the relationship between microstructure and groove geometries. It was observed that the higher KAM regions in the EBSD results correlated with the higher effective plastic deformation in the finite element analysis. Furthermore, it was inferred from the thermal cycle curves that variations in thermal cycles across different regions resulted differences in grain size and grain growth morphology.

## 1. Introduction

Invar 36 alloy, a single-phase austenitic alloy with extremely low coefficient of thermal expansion (CTE) between −200 °C and 200 °C, demonstrates unique application value in composite mold manufacturing, particularly as dimensional-stable molds for aircraft wing components [1,2,3]. In practical manufacturing, welding processes are essential for the integral forming of mold components and also serve as a critical technique for mold maintenance and repair [4,5,6]. Research confirms that the scientific optimization of weld groove geometries not only enhances joint performance but also reduces production costs [7,8,9,10].

Tungsten inert gas (TIG), MIG, and laser beam welding processes are widely employed in the joining of Invar 36 alloy [11]. However, conventional TIG and MIG welding of Invar 36 alloy is plagued by two primary challenges: solidification cracking and coefficient of CTE mismatch between the weld metal and base material [12,13]. Laser-MIG hybrid welding, as an advanced welding methodology, integrates the merits of two heat sources, combining the high penetration capacity of laser welding with the superior gap-bridging ability of arc welding [14,15]. In hybrid welding, the laser guiding effect on the arc significantly enhances arc constriction ratio, thus effectively improving arc stability [16]. Based on numerical simulations and experimental validation, Zhan et al. [17] found that Laser-MIG hybrid welding improves welding efficiency and reduces material consumption compared to conventional MIG welding. Their results also indicated that Laser-MIG hybrid welding provides improved welding stress distribution. Li et al. [18] compared the mechanical properties of Invar 36 joints made by laser-MIG hybrid welding and laser welding. Results showed that, compared with laser welding, the hybrid welding joints exhibited significantly higher yield strength and ultimate tensile strength, reaching 362 MPa and 473.5 MPa, respectively.

Furthermore, variations in welding grooves can modulate molten pool metal flow and heat dissipation, consequently altering the energy distribution within the molten pool. Zhou et al. [19] investigated laser-arc coupling during groove hybrid welding. They found that a laser-wire distance of 4 mm optimized this coupling, minimizing molten pool fluctuation and improving weld surface morphology. Gao et al. [20] explored the influence of groove parameters on weld formation and microstructure in laser–MIG hybrid welding, demonstrating that increasing the groove cross-sectional area mitigates the discrepancy between the laser-affected zone and arc-affected zone. Meng et al. [21] proposed that the rectangle groove and the Y-shape groove exhibit distinct spatial constraint effects (SCE) in laser–arc hybrid welding, where the spatial constraint of rectangle groove is significantly greater than that of Y-shape groove. This indicates that rectangle groove exhibits better thermal efficiency. However, there are limited reports on laser–MIG hybrid welding of Invar 36 alloy, and a notable gap exists in research on the microstructural evolution and mechanical property influence mechanisms under different weld grooves during laser–MIG hybrid welding, especially in multi-layer welding scenarios.

In this paper, three common types of grooves (V-shape, rectangle, and X-shape) were designed. Laser–MIG hybrid welding was performed on 10 mm thick Invar36 alloy plates. The effects of different welding bevels on the microstructure and mechanical properties of Invar36 alloy laser-MIG hybrid welded joints were investigated.

## 2. Materials and Methods

**Figure 1:**
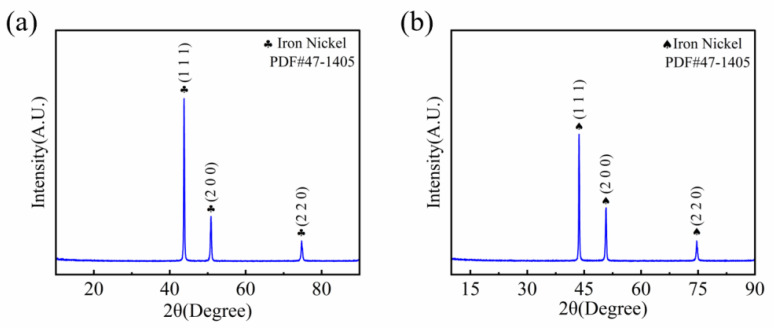
Characterization of (**a**) welding wire and (**b**) base metal.

The test material used was 10 mm-thick Invar 36 alloy plates, with dimensions of 80 × 80 × 10 mm. Three distinct groove configurations were adopted: V-shape, rectangle, and X-shape grooves for butt joint welding. The filler metal was 1.2 mm-diameter Invar 36 alloy welding wire. The X-ray diffraction (XRD) analysis results for both the base metal and welding wire, as depicted in Figure 1, revealed that both exhibit a face-centered cubic (FCC) crystal structure. Prior to welding, the groove and its surrounding area were ground with an angle grinder to remove the oxide layer, followed by decreasing with acetone to eliminate oil contamination.

**Figure 2:**
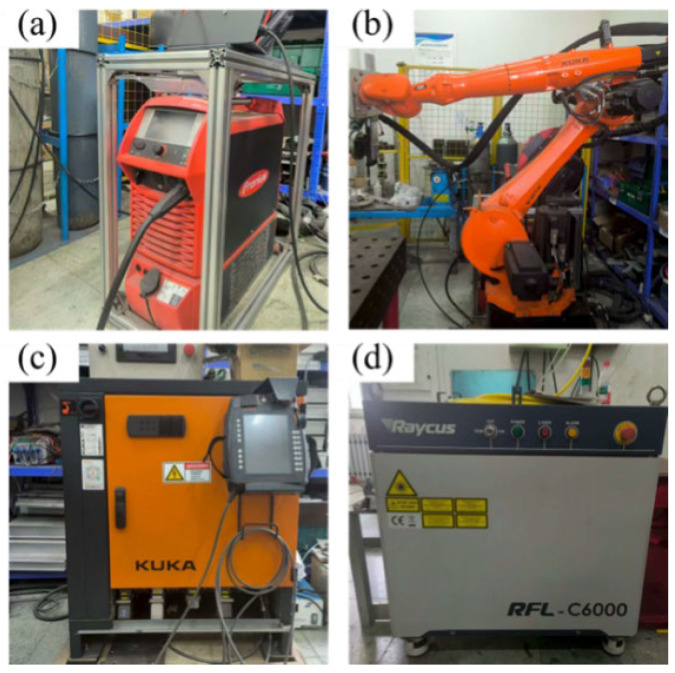
Laser–MIG hybrid welding device, (**a**) Lincoln Power Wave R350 arc welding system, (**b**) Control cabinet, (**c**) KUKA robot, and (**d**) IPG fiber laser.

**Figure 3:**
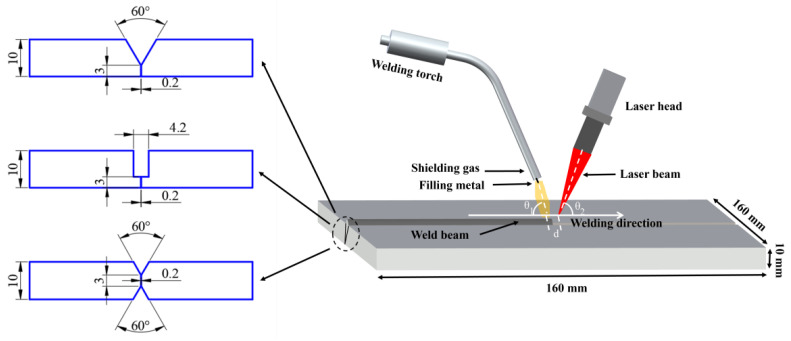
Schematic diagram of the welding.

As shown in Figure 2, the laser–MIG hybrid welding was conducted using a coupled system comprising IPG YLS-6000 fiber laser, Lincoln Power Wave R350 arc welding system, and KUKA robot. The primary parameters of the laser system are as follows: a maximum output power of 6 kW, a wavelength of 1.07 μm, a beam parameter product of 12 mm · mrad, a transmission fiber core diameter of 200 μm, a beam diameter of 0.38 mm, and operation in continuous-wave (CW) mode. Arc welding system supports multiple welding modes and features an integrated process database that automatically adjusts voltage and wire feed speed according to the user-defined welding current. As illustrated in Figure 3, the welding torch was inclined at a tilt angle θ_1_ of 70° from the horizontal, while the laser beam was angled at θ_2_ = 85° to prevent laser reflection from damaging optical components. During the welding process, the laser beam preceded the welding torch with a laser-wire distance set at 2 mm. Argon gas with a purity of 99.99% was used as the shielding gas, delivered at a flow rate of 20 L/min. The welding process was divided into two layers: root pass and filling pass.

To ensure complete penetration through the 3 mm root face in the grooves, the root pass parameters were set as laser power 3500 W, welding current 240 A, and welding speed 1.3 m/min. Considering that the filler pass requires a greater metal deposition rate and excessive heat input can degrade weld quality, the laser power was reduced, and the travel speed was decreased for this pass. The welding current was maintained as constant to ensure a stable welding process. The filling pass parameters were set as laser power 2500 W, welding current 240 A, and welding speed 1 m/min. Given the symmetry of the X-shape groove and to minimize the number of variables when comparing it with other groove types, the same parameters were used on both sides: laser power 3500 W, welding current 240 A, and welding speed 1 m/min.

Metallographic specimens were sectioned from the welded joint cross-sections using wire electrical discharge machining (WEDM), followed by mechanical grinding, polishing, and etching with a 6% nital solution (nitric acid in alcohol). The microstructure of the weld joint was observed using an OLYMPUS-DSX510 optical microscope (OM). Grain morphology and orientation in the joint were analyzed via field-emission scanning electron microscopy (FE-SEM, MERLIN Compact, Zeiss) and electron backscatter diffraction (EBSD). The EBSD data were processed and analyzed using the OIM Analysis 7 software.

**Figure 4:**
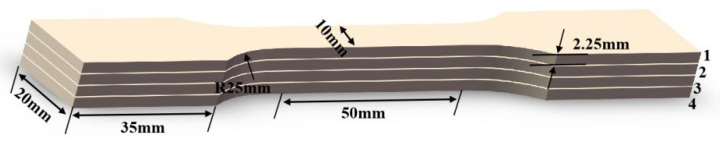
Schematic diagram of tensile specimens.

Microhardness testing across different regions of the weld joint was executed using a microhardness tester. The test positions were set 2 mm above the weld center designated as the top region and 2 mm below the weld center designated as the bottom region. Microhardness testing across different regions of the weld joint was executed using a microhardness tester. A maximum load of 300 g was applied during the microhardness test, with a holding time of 15 s at the maximum load. A universal testing machine was utilized to carry out mechanical property tests on the welded specimens, with a tensile rate set at 0.5 mm/min. Given the distinct welding parameters for each layer and the heat treatment effect of the second weld pass on the first, interlayer sampling was conducted at different positions within the weld to investigate the performance of each layer. The dimensions of the tensile specimens are illustrated in Figure 4. For each groove type, three tensile specimens were prepared. The specimen exhibiting the median mechanical properties from each set was selected for stress–strain curve plotting. The average tensile strength was calculated based on the data from all twelve specimens.

This experiment conducted finite element analysis via Abaqus 2020 to predict residual stress and temperature distribution. A finer mesh was located at the weld zone to ensure the simulation precision, and a coarse mesh was applied at the area far from the weld bead to reduce the computational cost. In this experiment, finite element models were established for each type of groove. The V-shape groove comprised 120,640 nodes and 11,682 elements. Rectangle groove consisted of 131,562 nodes and 126,418 elements. The X-shape groove included 124,012 nodes and 120,082 elements. All models were using C3D8RT elements (eight node hexahedral). The initial environmental condition was set to 20 °C. Rigid constraints were imposed on both sides of the model. The material parameters were referred to the research of Zhan et al. [8,22]. Convection and radiation were applied to the surfaces of the finite element model, while rigid constraints were imposed on both sides of the model:

(1) $$K_{n}\frac{\partial T}{\partial n}+Q+h(T_{0}-T)+\varepsilon \sigma_{SB}(T_{0}^{4}-T)=0$$

*K_n_* is thermal conductivity normal to the surface, *Q* represents the prescribed flux, h is heat transfer coefficient for convection, *σ_SB_* is Stefan-Boltzmann constant, *ε* denotes the emissivity for surface radiation (0.5), and *T*_0_ is the ambient temperature for convection and radiation (20 °C). The element birth and death techniques were employed to simulate multi-pass welding. Given that the laser–arc hybrid welding involves dual heat sources, the double-ellipsoid heat source was selected to model the arc heat source, while the combined conical and cylindrical shell heat sources were chosen to represent the laser heat source model. The formula for the double-ellipsoid heat source is as follows:

(2) $$q_{f}=\frac{6\sqrt{3}UIe}{a_{f}bc\pi \sqrt{\pi }}\exp(-3\frac{x^{2}}{a_{r}^{2}}-3\frac{y^{2}}{b^{2}}-3\frac{z^{2}}{c^{2}})$$

(3) $$q_{r}=\frac{6\sqrt{3}UIe}{a_{r}bc\pi \sqrt{\pi }}\exp(-3\frac{x^{2}}{a_{r}^{2}}-3\frac{y^{2}}{b^{2}}-3\frac{z^{2}}{c^{2}})$$

*q_f_* are the heat input of the forward and backward ellipsoid heat sources, *U* is voltage, *I* is current, and $a_{r}$, $a_{f}$, *b*, *c* are constants. The formula for the combined conical and cylindrical shell heat source is as follows:

(4) $$q_{l1}=\frac{9\eta Pe^{3}}{\pi (e^{3}-1)(z_{i}-z_{e})(r_{i}^{2}+r_{i}r_{e}+r_{e}^{2})}\exp(-3\frac{3(x^{2}+y^{2})}{r_{1}})$$

(5) $$q_{l2}=\frac{9\eta Pe^{3}}{\pi (e^{3}-1)(z_{t}-z_{i})(r_{t}^{2}+r_{i}r_{t}+r_{i}^{2})}\exp(-3\frac{3(x^{2}+y^{2})}{r_{2}})$$

(6) $$r_{1}=r_{i}-\frac{\left(r_{i}-r_{e})(z_{i}-z\right)}{z_{i}-z_{e}}$$

(7) $$r_{2}=r_{t}-\frac{\left(r_{t}-r_{i})(z_{t}-z\right)}{z_{t}-z_{i}}$$

*q_l_*_1_, *q_l_*_2_ is the heat input of the combined conical and cylindrical shell heat source, *r*_1_, *r*_2_ are distribution parameters and *z_i_*, *z_e_*, *z_t_*, *r_i_*, *r_e_*, *r_t_* are constants.

## 3. Results and Discussion

### 3.1. Macroscopic Morphology and Microstructure

**Figure 5:**
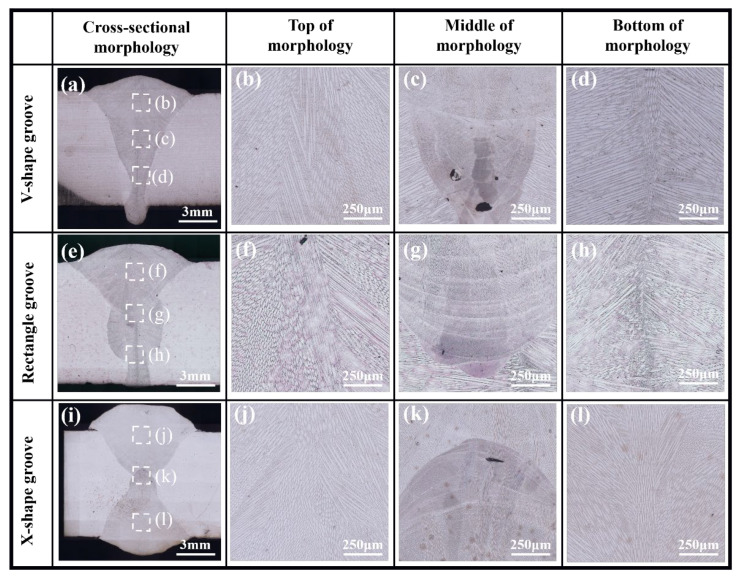
Cross-section morphologies of different grooves: (**a**) V-shape groove, (**b**–**d**) local enlarged view of V-shape groove; (**e**) rectangle groove, (**f**–**h**) local enlarged view of rectangle groove; (**i**) X-shape groove, and (**j**–**l**) local enlarged view of X-shape groove.

In the three types of weld joints, the welds were divided into upper and lower layers, which were shown in Figure 5a,e,i. But in the X-shape groove, the weld displayed a symmetrical upper-lower distribution. Small-sized pores were observed in Figure 5, attributed to keyhole instability that causes local expansion and bulging of the front and rear keyhole walls. This instability led to liquid metal blockage at the keyhole bottom, followed by root closure forming a cavity. The high viscosity of liquid Invar 36 alloy hindered gas escape, ultimately resulting in pore formation at the molten pool bottom [23].

Distinct thermal behaviors exhibited by different welding grooves exert significant influences on the weld microstructure during the cooling phase [24]. Figure 5 displayed the cross-sectional views of welds under different groove types, indicating that columnar grains in the weld grew from both sides toward the center. The reason was that the morphology of grains during molten pool solidification was determined by the competitive growth process. Under different solidification conditions, the molten pool exhibited varying growth directions. When columnar grains growth direction coincides with the maximum temperature gradient, columnar grained a growth advantage, thus the final growth direction of columnar grains was close to the maximum temperature gradient [25]. Additionally, vertical columnar grains were clear at the center of both V-shape groove and X-shape groove welds. The phenomenon resulted from the high heat input in the laser–arc hybrid zone, which altered the direction of the maximum temperature gradient [26].

**Figure 6:**
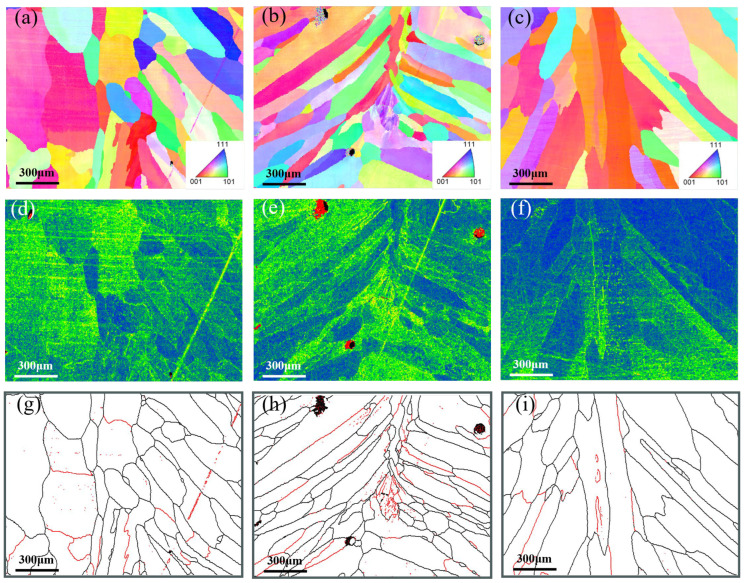
V-shape groove: (**a**–**c**) IPF of different regions (top, middle, and bottom), (**d**–**f**) KAM of different region, and (**g**–**i**) different region of Grain boundary maps.

**Figure 7:**
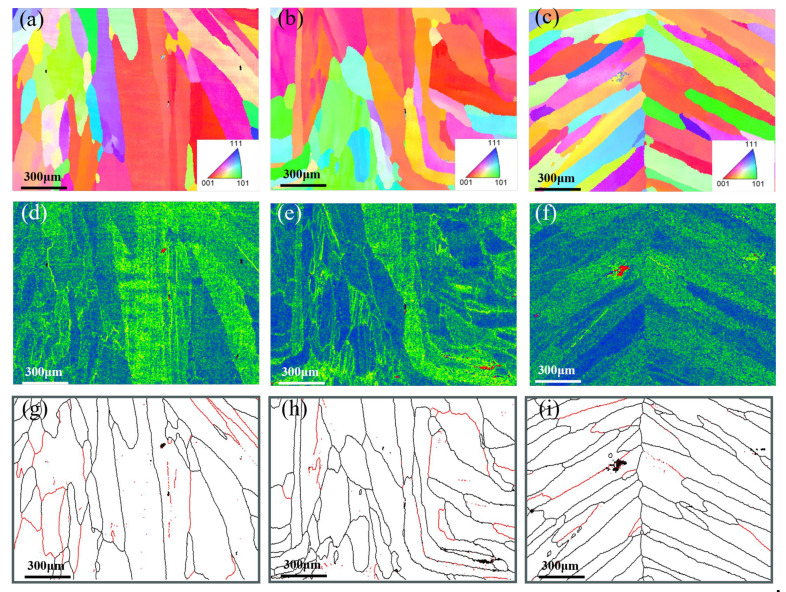
Rectangle groove: (**a**–**c**) IPF of different regions (top, middle, and bottom), (**d**–**f**) KAM of different region, and (**g**–**i**) different region of Grain boundary maps.

**Figure 8:**
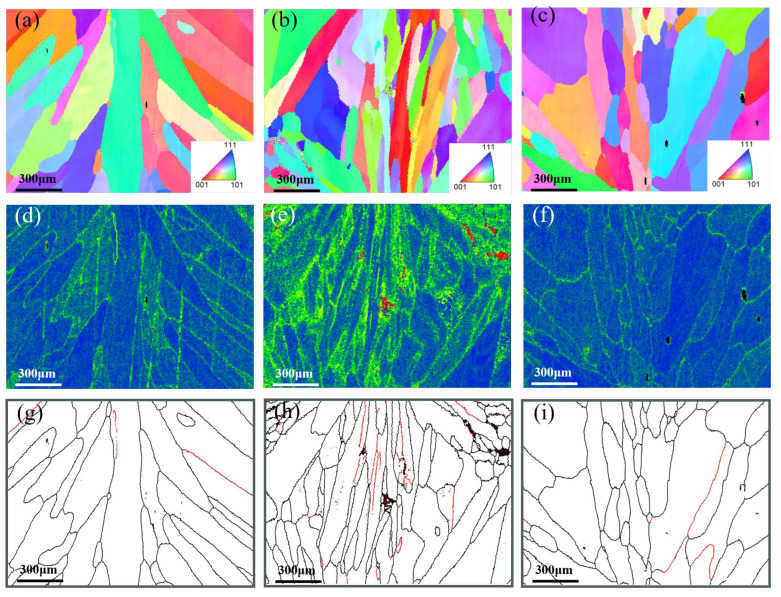
X-shape groove: (**a**–**c**) IPF of different regions (top, middle, and bottom), (**d**–**f**) KAM of different region, and (**g**–**i**) different region of Grain boundary maps.

EBSD analysis results for the root pass, the junction between root pass and fill pass, and the fill pass regions of different groove types were shown in Figure 6, Figure 7 and Figure 8. The measurement positions corresponded to the local enlarged views in Figure 5. Figure 6a–c, Figure 7a–c and Figure 8a–c presented Inverse Pole Figure (IPF) maps of the weld zones, showing that grain structures across groove types are similar. Weld columnar grains grew from both sides toward the center. The grains in the top region were the most course, in the middle region were the finest, and the bottom region grains were intermediate in size. Since the middle region of the weld was the laser zone of the second weld pass, where a higher temperature gradient existed. This promoted the formation of abundant crystal nuclei perpendicular to the molten pool boundary, which subsequently grew and intersected with adjacent columnar grains. Figure 6f–h, Figure 7f–h and Figure 8f–h showed the Kernel Average Misorientation (KAM) maps of the welds. Higher KAM values corresponded to greater lattice strain and elevated dislocation density. The highest KAM values were observed at the weld center due to its final solidification, which induced significant residual stresses [27]. Figure 6i–k, Figure 7i–k and Figure 8i–k showed the grain boundary maps of the weld center. In these maps, black lines represented high-angle grain boundaries (HAGB, 15–60°) and red lines denote low-angle grain boundaries (LAGB, 3–5°). It was evident that the middle region of the weld exhibited a higher density of LAGBs, while the top and bottom regions contained fewer LAGBs.

As shown in Figure 6a, the weld top region exhibited the coarsest grains and cast structure characteristics because the higher heat input in the second weld pass prolonged the residence time of the molten pool at the top. The middle region of the weld was the intersection of the laser zone from the second pass and the arc zone from the first pass. Due to the concentrated laser energy density, a large temperature gradient existed during cooling, thus resulting in fine grains. Additionally, the middle region of Figure 6e showed higher KAM values, and Figure 6h contained more low-angle grain boundaries (LAGBs), indicating a high degree of plastic deformation and high dislocation density in this zone [28]. As shown in Figure 7a, the change in groove geometry made modification of the molten pool shape, thus leading to a higher density of perpendicularly oriented grains in the weld. As depicted in Figure 7b, the lower right grains displayed an “L”-shaped growth pattern, whereas the lower left grains were mixed columnar and equiaxed grains. The formation of these special-shaped grains arose from the abrupt change in temperature gradient direction as columnar grains solidify toward the weld center, causing a shift in crystal growth orientation. This morphological feature affected weld performance by inducing stress concentration during tensile loading, potentially leading to weld failure [29]. Figure 7c showed that the perpendicular columnar grains in the weld disappear, with grains growing laterally from the centerline. This crystal structure featured lower stress but reduced tensile strength, making cracks prone to propagate at the weld center. As seen in Figure 8a,c, the weld showed top-bottom symmetry and perpendicular columnar grains interleaved with laterally growing columnar grains, which were observed in Figure 8d. The higher KAM values and abundant low-angle grain boundaries were in this region, which were typical of high dislocation density, suggested that the weld middle had high tensile strength [30].

**Figure 9:**
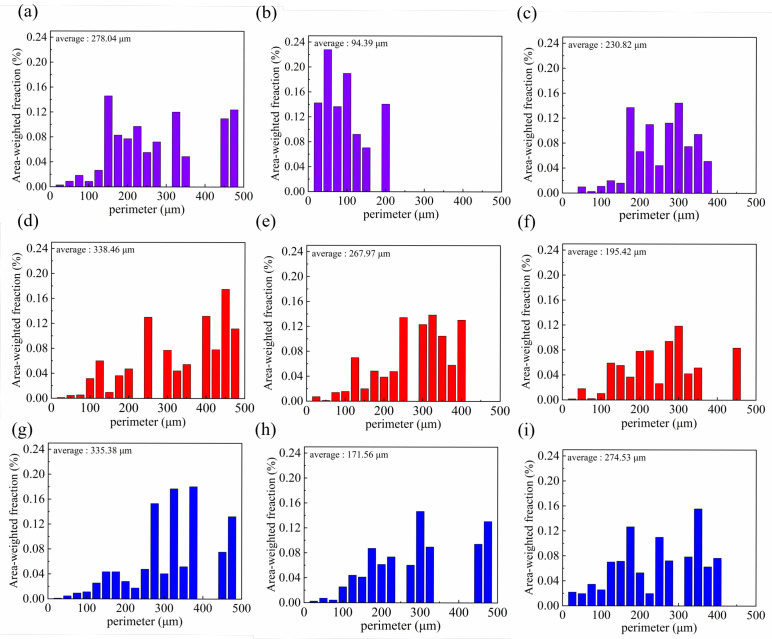
Statistical plots of grain size for EBSD results of different grooves: (**a**–**c**) Different regions (top, middle, and bottom) of the V-shape groove, (**d**–**f**) different regions (top, middle, and bottom) of the rectangle groove, (**g**–**i**) different regions (top, middle, and bottom) of the X-shape groove.

Figure 9 showed the grain size statistics of different welding grooves, corresponding to Figure 6, Figure 7 and Figure 8. The V-shape groove exhibited the finest grain size, while the rectangle and X-shape grooves had similar grain sizes. The grain size in the weld center was significantly smaller than that in the top and bottom regions. In the V-shape and rectangle grooves, the dimensions of the grain of the first weld pass were obviously smaller than the top region of the second pass.

**Figure 10:**
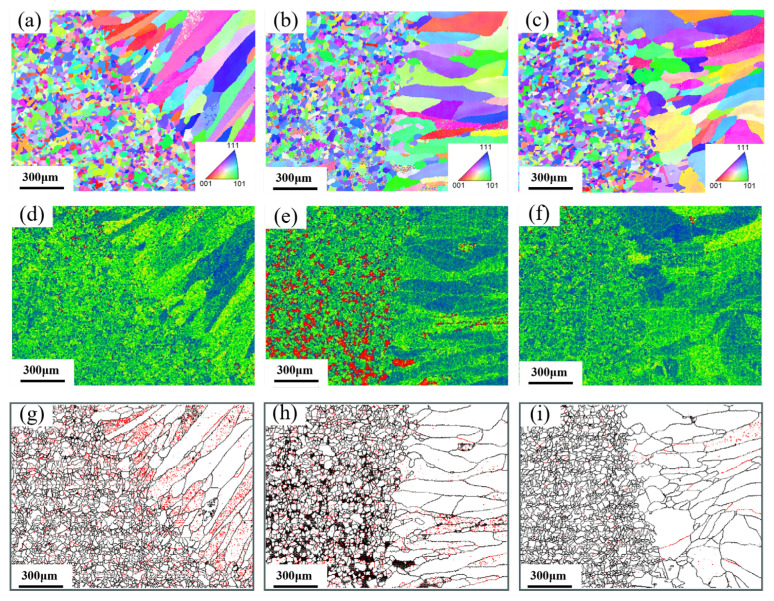
(**a**–**c**) IPF at the interface between base metal and weld metal for different grooves, (**d**–**f**) KAM of different grooves, and (**g**–**i**) different grooves of Grain boundary maps.

In Figure 10, the fine equiaxed grains on the left denoted the base metal, while the coarse columnar grains on the right represented the weld zone. Due to the high heat input during welding, the cooling rate in the molten pool center was slow, leading to coarse grain formation in the weld zone. By contrast, grain changes in the heat-affected zone (HAZ) were insignificant, confirming that the weld center was a weak region. Figure 9d–f presented the KAM maps for the corresponding regions. The base metal exhibited higher KAM values due to its rolled and annealed microstructure, whereas the KAM values in the weld regions of different grooves were similar and lower than those in the base metal [31]. Referring to Figure 10d–f, the KAM value distributions in the corresponding regions indicated that the base metal exhibited significantly higher KAM values than typical recrystallized microstructures due to residual plastic deformation from its rolled and annealed texture. It is worth emphasizing that the KAM values in the weld regions of all groove types showed similar levels. Compared to Figure 6, Figure 7 and Figure 8, it presented a high value like Figure 6e, Figure 7e and Figure 8e, both exceeding those in the weld center areas depicted in Figure 6d,f, Figure 7d,f and Figure 8d,f.

**Figure 11:**
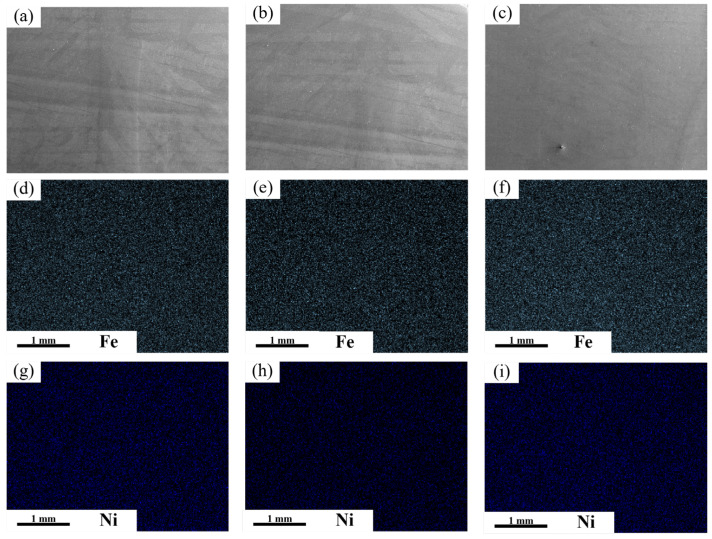
(**a**–**c**) V-shape groove SEM images of different regions (top, middle, and bottom), corresponding element mapping results of different regions: Fe: (**d**–**f**) and Ni: (**g**–**i**).

As shown in Figure 11, EDS area scans and point scans were performed on the upper, middle, and lower regions of the weld seam. The results revealed no significant fluctuation in chemical composition. As depicted in Figure 11c,f,i, no discernible difference in composition was observed between the base metal and the weld metal.

### 3.2. Mechanical Properties

**Figure 12:**
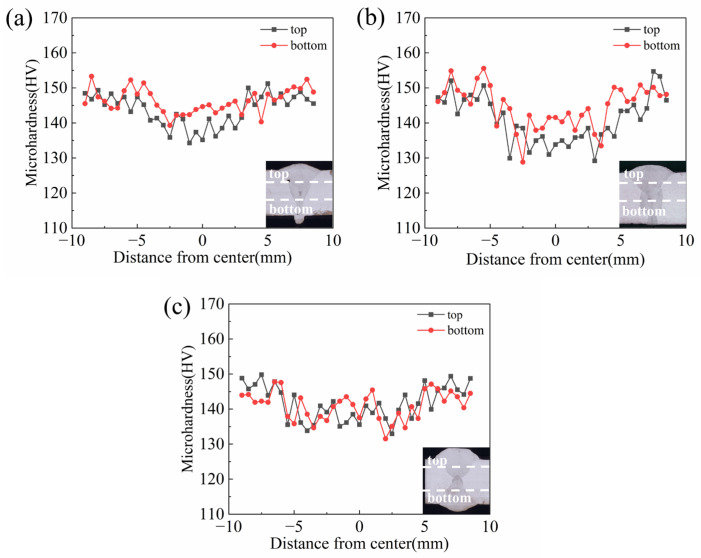
Microhardness distribution of different grooves: (**a**) V-shape groove, (**b**) rectangle groove, and (**c**) X-shape groove.

In Figure 12, the microhardness profiles of V-shape, rectangle, and X-shape grooves were displayed. The results revealed that the V-groove had the highest microhardness, followed by the X-groove, and the rectangle groove showed the lowest value. In both V-shape and rectangle grooves, it was obvious to see that microhardness was different between the top and bottom regions, with the bottom manifesting as notably higher hardness. This was strongly correlated with the finer grain size at the bottom compared to the top. Hardness testing revealed no softening characteristics in the HAZ.

**Figure 13:**
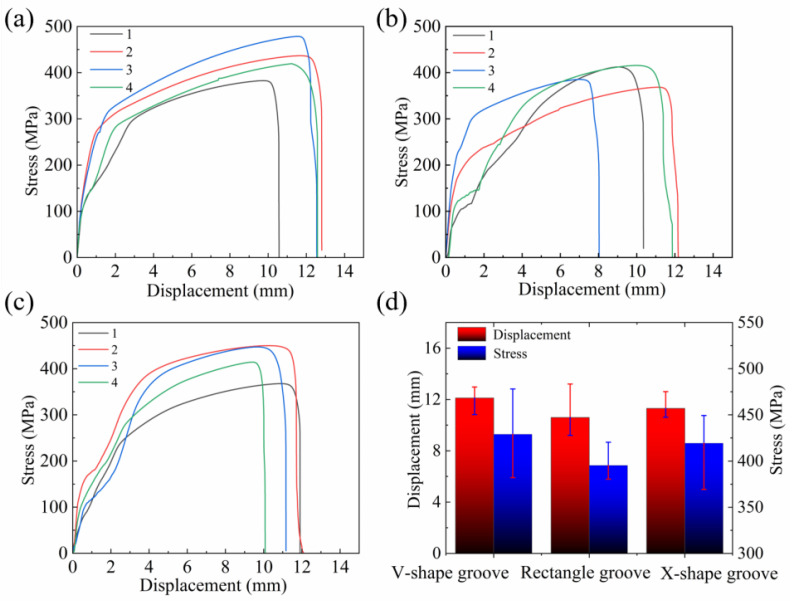
Mechanical properties of welded joints under different grooves: stress-displacement curves for different interlayer locations of (**a**) V-shape, (**b**) rectangle, (**c**) X-shape grooves, and (**d**) average mechanical properties of all specimens.

The stress-displacement curves for different interlayer locations of the V-shape, rectangle, and X-shape grooves were shown in Figure 13a, Figure 13b and Figure 13c, respectively. Figure 13a revealed that in the V-shape groove, tensile specimens 2 and 3 positioned in the middle region shown higher tensile properties, with ultimate tensile strengths (UTS) reaching 435.9 MPa and 478.1 MPa, respectively. By contrast, specimen 1 showed a notably lower UTS of 382.6 MPa. This was because specimen 1 was in the arc zone of the second weld pass, manifesting coarse grains, while specimens 2 and 3 corresponded to the region near the weld center with fine grains, which aligned with the EBSD results. Figure 13b demonstrated that rectangle groove specimen 3 had the lowest UTS of 385.5 MPa. This was attributed to the grain morphology observed in Figure 13c. Additionally, it was observed in Figure 5i that there existed grains parallel to the direction of tensile stress, which may cause crack propagation from the weld center, thereby leading to weld failure. In the X-shape groove weld, the weld center demonstrated superior mechanical properties, with specimens 2 and 3 each having UTS values of 449.8 MPa and 447.0 MPa. By contrast, the surface and bottom layers exhibited inferior properties, as specimens 1 and 4 showed UTS values of 367.7 MPa and 413.8 MPa, as depicted in Figure 13c. The reason was that the weld center in the X-shape groove featured small-sized columnar grains with interlaced distribution, while the grain size in the surface and bottom layers was significantly larger than that in the center. Additionally, the high KAM values at the weld center induced greater lattice distortion, higher dislocation density, and enhanced tensile strength.

**Figure 14:**
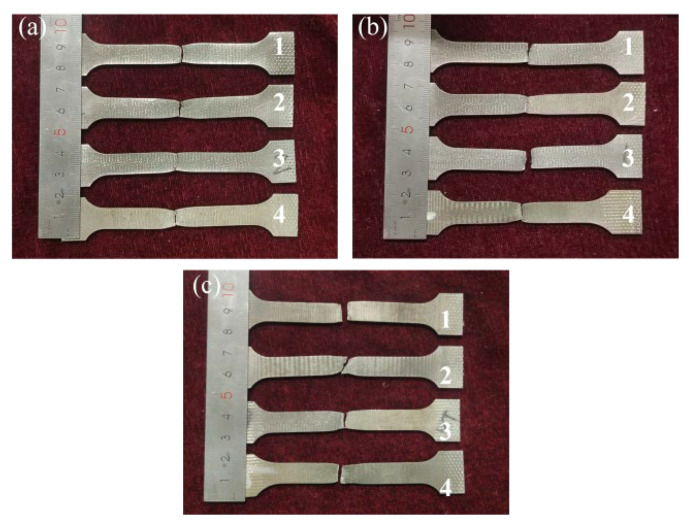
Schematic diagram of fracture location in tensile specimen: (**a**) V-shape groove, (**b**) rectangle groove, and (**c**) X-shape groove.

Figure 13d presented the average tensile strength and displacement for different grooves of all specimens. The V-groove exhibited the highest average ultimate tensile strength (UTS) of 429.0 MPa, followed by the X-shape groove at 419.3 MPa, and the rectangle groove at 395.4 MPa. This stemmed from fewer welding defects in the welds, with performance significantly correlating with grain size and morphology [32]. As illustrated in Figure 14, the tensile fracture location maps for different grooves indicated that all fractures occurred at or near the weld center. Notably, every tensile specimen developed a distinct necking after the test.

### 3.3. Thermal Mechanical History

**Figure 15:**
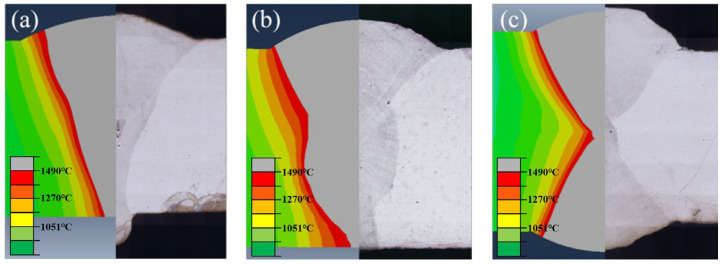
Comparison of experimental and numerical weld cross-section: (**a**) V-shape groove, (**b**) rectangle groove, and (**c**) X-shape groove.

The peak temperature distributions of welded joints with different grooves, as predicted by Abaqus, were presented in Figure 15. The validity of a finite element simulation is generally based on two criteria: good agreement between the simulated and actual macroscopic profiles, and accurate capture of key characteristics [33,34,35]. The macroscopic profile simulated by the coupled thermal model as slightly wider than that of the experimental results. The results showed that the simulation results can reliably predict the macroscopic profile of the weld to a certain extent.

**Figure 16:**
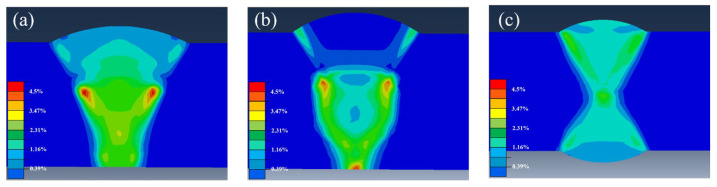
The effective plastic strain of welded joints under different grooves: (**a**) V-shape groove, (**b**) rectangle groove, and (**c**) X-shape groove.

Figure 16 depicted the predicted distribution of effective plastic strain (PEEQ) history for different grooves, which represented the average plastic deformation at the element integration points in the finite element model [36]. The simulation results were consistent with the work by He et al., with the highest value appearing at the junction between the weld and the HAZ [37]. The values of effective plastic strain (PEEQ) exhibited significant differences across the various grooves. As shown in the results, the X-shape groove demonstrated a lower PEEQ value compared to both the rectangle and V-shape groove. This lower PEEQ value suggested reduced levels of residual stress within the X-groove weld joint. Furthermore, distinct PEEQ values were also observed between the V-shape and X-shape grooves. This difference is attributed to the inherent variations in their geometric configurations. The data indicated that the strain value in the weld was higher at the mid region, and the highest strain value was observed near the HAZ within the weld. Thus, it was inferred that the reason KAM values in Figure 10 were higher than those in Figure 6d,f, Figure 7d,f and Figure 8d,f was that significant plastic deformation occurs near HAZ because of residual stress. This deformation generated many dislocations, inducing high micro-stress that promoted an increase in KAM values. And the high value in Figure 6e, Figure 7e and Figure 8e was caused by the weld center undergoing the fastest cooling during solidification and being subjected to the strongest triaxial constraints.

**Figure 17:**
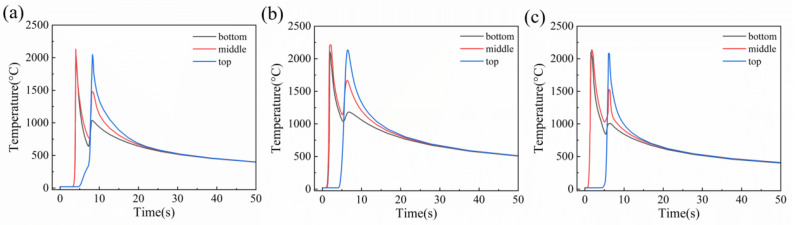
Thermal cycle curves at different welding groove positions: (**a**) V-shape groove, (**b**) rectangle groove, and (**c**) X-shape groove.

As illustrated in Figure 17, the thermal cycle diagrams corresponding to various groove regions were presented. Table 1 showed the fitting cooling rates at different positions of different grooves. The relationship between cooling rate and grain size demonstrated a significant negative correlation. In both V-shape and rectangle grooves, the thermal cycle curves at the bottom and top of the weld showed a high degree of similarity. Notably, the cooling rate of the first weld pass within the weld was significantly higher than the second weld pass. Consequently, the grain size of the first weld pass was smaller than its counterpart. Furthermore, it was observed that the cooling rate and temperature gradient during the cooling at the bottom of the weld surpassed its top [38,39]. Since the heat dissipation coefficient of metals was much higher than that of air, the bottom of the thick plate achieved excellent heat dissipation [40]. Therefore, the cooling rate exhibited an increasing trend along the penetration direction, further influencing the microstructure and mechanical properties. And that was why the grains in the first weld pass were finer than those in the second weld pass. For the X-shape groove, the cooling curves at the top and bottom were basically the same. Temperature measurements revealed that the cooling rate of the second weld pass in the X-shape grooves was significantly lower than those observed in both the V-shape groove and the rectangle grooves.

### 3.4. Implications for Practical Applications and Future Work

The findings of this study provided feasible strategies for selecting groove geometries in the laser–MIG hybrid welding of 10 mm thick Invar36 alloy plates, which was critical for manufacturing large-scale, dimensionally stable molds in aerospace and other high-precision industries.

The V-shape groove yielded the highest tensile strength (429.0 MPa) and favorable microhardness distribution. This was attributed to the presence of vertical columnar grains at the weld center, which effectively hindered crack propagation. Therefore, for critical structural applications where joint strength is the paramount concern, such as the primary load-bearing components of composite molds, the V-shape groove is highly recommended.

Rectangle groove showed the lowest mechanical properties among the three, primarily due to the absence of strengthening vertical columnar grains and the presence of grains parallel to the stress direction. However, its simple geometry offers the advantage of ease of preparation and lower cost. Its application could be considered for structures where moderate strength is acceptable, and cost-effectiveness is a priority. Additionally, for future studies, modifying rectangle groove with a small root gap might improve molten pool dynamics and grain morphology.

The X-shape groove, while exhibiting a slightly lower average strength (419.3 MPa) than the V-shape groove, demonstrated a symmetrical thermal cycle and microstructure. This symmetry is advantageous for controlling welding distortion in long-seam welds. Although it requires welding from both sides, potentially increasing process time, the balanced heat input minimizes angular distortion, which is crucial for maintaining the dimensional accuracy of large molds. Thus, for long welds where controlling distortion is as important as strength, the X-shape groove presents a balanced choice.

Based on the findings of this study, future research should focus on a more detailed optimization of groove parameters. Furthermore, given the observed pores in Invar36 alloy, it is critical to quantitatively analyze the influence of groove geometry on molten pool flow dynamics and gas escape behavior, with the aim of establishing groove designs that effectively minimize porosity rates in gas-sensitive alloys.

## 4. Conclusions

Laser–MIG hybrid welding experiments were performed on 10 mm thick Invar36 alloy plates. The influence of three different types of welding grooves (V-shape, rectangle, and X-shape) on the microstructure and mechanical properties of the welded joints were analyzed:

1. Distinct thermal behaviors exhibited by different welding grooves exerted significant influences on the weld microstructure during the cooling phase. In the V-shape and X-shape grooves, vertical columnar grains were found between the columnar grains that grow toward both sides, whereas vertically growing columnar grains were absent in rectangle groove. In all three groove types, the finest grains were located at the weld center. While the V-shape and rectangle grooves exhibit coarser grains at the top region, the grain size distribution in X-shaped grooves was symmetric between the top and bottom.
2. The highest average tensile strength for the V-shape groove, X-shape groove, and rectangle groove were 429.0 MPa, 419.3 MPa, and 395.4 MPa, respectively. The tensile properties of the laser zone in the weld were significantly better than those of the arc zone. Vertical columnar grains in the weld enhanced the tensile strength of the weld. The weld center in the grooves featured small-sized columnar grains with interlaced distribution, while the grain size in the surface and bottom layers was significantly larger than that in the center. Additionally, the high KAM values at the weld center induced greater lattice distortion, higher dislocation density, and enhanced tensile strength.
3. The results of finite element analyses revealed that regions with high plastic deformation correspond to areas of high KAM values in the weld, and the relationship between cooling rate and grain size was explained through thermal cycle curves: regions with faster cooling rates clearly exhibit finer grain structures.
4. The influence of the groove configuration on performance primarily stems from differences in grain morphology and residual stresses. The V-shape groove is recommended for critical structures requiring ultimate strength, for long welds where controlling distortion is as important as strength. The X-shape groove is preferred for long welds to minimize distortion, whereas rectangular groove offers a cost-effective solution for non-critical components.

## Figures and Tables

**Table 1:** The fitting cooling rates at different positions of different grooves (°C/s).

Region	V	Rectangle	X
Top	270.6	225.85	357.79
Bottom	540.92	325.31	334.36

## Data Availability

The original contributions presented in this study are included in the article. Further inquiries can be directed to the corresponding author.
